# FROM KNOWING TO DOING: WHAT IS NEEDED TO SUPPORT PATIENTS IN CHANGING PHYSICAL ACTIVITY

**DOI:** 10.1093/geroni/igac059.065

**Published:** 2022-12-20

**Authors:** Anne Thackeray, Tonua Hamilton, Janice Morse, Rachel Hess, Molly Conroy, Julie Fritz

**Affiliations:** University of Utah, Salt Lake City, Utah, United States; University of Utah, Salt Lake City, Utah, United States; University of Utah, Salt Lake City, Utah, United States; University of Utah, Salt Lake City, Utah, United States; University of utah, Salt Lake City, Utah, United States; University of Utah, Salt Lake City, Utah, United States

## Abstract

Physical therapists often treat pain and functional limitations associated with chronic musculoskeletal conditions common in aging adults. While patient report improvement after physical therapy, these results do not translate to sustained physical activity. This is a lost opportunity to support aging adults in adopting behaviors proven to improve quality of life and reduce comorbidity burden. We conducted semi-structured interviews with 30 physical therapists to understand how they support adoption of physical activity and identify what is needed to improve uptake. Physical therapists endorse physical activity as essential in the management of MSK conditions. Eliciting motivation, addressing psychosocial needs, and empowering patients to actively engage in solutions were identified as significant challenges in the effort to change physical activity. At the clinician level, physical therapists identified the need for improved skills in motivational interviewing and person-centered communication. Improved coordination with mental health providers and community resources were identified as environmental needs.

